# The Diagnostic Value of Serum Ang, VEGF, and CRP Combined with the Chinese Medicine Antitumor Formula in the Treatment of Advanced Renal Carcinoma

**DOI:** 10.1155/2021/5189069

**Published:** 2021-12-14

**Authors:** Nana Dong, Shengmin Zhang, Shuangjun Zhang, Qiongqiong Zhao, Donghua Zhang, Feng Chen

**Affiliations:** ^1^Department of Oncology (I), Yantaishan Hospital, Yantai 264000, China; ^2^PIVAS, Qingdao Central Hospital, Qingdao University, Qingdao 266000, China; ^3^Department of Imaging, Zhangqiu District People's Hospital, Jinan 250200, China; ^4^Department of Paediatrics (II), Zhangqiu District People's Hospital, Jinan 250200, China; ^5^Tumor-Chemotherapy Department, Zhangqiu District People's Hospital, Jinan 250200, China; ^6^Internal Medicine Department, Weifang Municipal Government Hospital, Jinan 250200, China

## Abstract

**Objective:**

To explore the diagnostic value of serum angiopoietin (Ang), vascular endothelial growth factor (VEGF), and C-reactive protein (CRP) combined with the Chinese medicine antitumor formula in the treatment of advanced renal carcinoma.

**Methods:**

Retrospective analysis was performed for the data of 60 patients with advanced renal cancer admitted at Yantaishan Hospital from February 2019 to February 2020. All patients were treated with Chinese medicine antitumor formula. The serum Ang, VEGF, and CRP levels in venous blood samples were detected before and after treatment. Sensitivity, specificity, and AUC of combined serum Ang, VEGF, and CRP were analyzed utilizing the receiver operating characteristic curve (ROC) (95% CI).

**Results:**

There were 52 cases of clear-cell carcinoma (86.7%), 7 cases of papillary carcinoma (11.7%), and 1 case of chromophobe renal cell carcinoma (1.7%). The average tumor diameter was (9.67 ± 0.65) cm, and the KPS score was (74.68 ± 1.52). About 75% of the patients had metastasis. After treatment, the level of serum Ang, VEGF, and CRP was immensely lower compared to that before treatment (*P* < 0.001). The sensitivity, specificity, and AUC (95%CI) of the combined detection of Ang, VEGF, and CRP before treatment were 86.7%, 90.0%, and 0.883 (0.817–0.950), while the sensitivity, specificity, and AUC (95%CI) of the combined detection of Ang, VEGF, and CRP were 83.3%, 86.7%, and 0.850 (0.776–0.9524), respectively.

**Conclusion:**

The combined detection of serum Ang, VEGF, and CRP has high diagnostic value for patients with advanced renal cancer treated with Chinese medicine antitumor formula.

## 1. Introduction

Renal cancer is a malignant tumor that occurs in the urinary tubule epithelium of the renal parenchyma. The incidence rate of the disease in 2011 was 3.8%. According to the data of the Ministry of Health of China, its incidence has been increasing year by year in the past ten years and now ranks top ten among the highest incidence of male malignant tumors in China [1, 2]. Since the early symptoms of kidney cancer are not specific, most patients are being found in the middle and late stages of the disease when they are diagnosed [3]. Patients with advanced metastatic renal cancer lack sensitivity to conventional treatments such as radiotherapy and chemotherapy [4]. Their average life expectancy is less than 1 year, and the five-year survival rate is less than 10% [5–7], and patients usually have a poor prognosis. Prolonging the survival time of patients with advanced renal cell carcinoma is the focus of clinical research. However, no optimum remedies for renal cancer have been found at present, and some studies have shown that traditional Chinese medicine (TCM) has unique advantages in the treatment of malignant urinary tumors [8–10]. Since renal cancer is an immune-related malignant tumor, the application of TCM treatment based on the concept of holistic treatment and syndrome differentiation is beneficial to improve the body tolerance of patients and control the progression of renal cancer. The findings of the previous study show that the Chinese medicine antitumor formula has a substantial targeted effect on advanced cancer [11]. The prescription contains yangtao actinidia root, Fructus Akebiae, Herba Solani Lyrati, and Catechu, which can effectively reduce the level of inflammatory factors in patients, enhance their immunity, inhibit the expression of VEGF and its receptors, reduce the density of microvessels in tumor tissues, play a role in blocking the process of the cancer cell cycle, and prolong the survival period of patients with advanced cancer. However, few studies have used the Chinese medicine antitumor formula to treat renal cancer, and the drug's actual effect on patients with advanced renal cancer is still unclear. Therefore, when applying the Chinese medicine antitumor formula, attention should be paid to monitoring the patient's disease progression to ensure the patient gets the highest therapeutic benefit.

At present, there is a lack of ideal markers for diagnosing advanced renal cancer in clinical practice. For renal cancer patients treated with the Chinese medicine antitumor formula, an index with high consistency with the mechanism of action should be selected. VEGF is an important indicator that reflects the ability of blood vessel proliferation, and CRP is closely related to the level of inflammation in patients [12–14]. In addition to VEGF, Ang is also closely associated with angiogenesis, and Ang-1 and Ang-2 in this protein family belong to angiogenesis regulators. Numerous studies have confirmed that their expression levels are closely related to tumor stages and patient prognosis [15, 16]. Therefore, Ang, VEGF, and CRP were selected as the serological indicators to explore the value of their combined detection for patients with advanced renal cancer after treating with the Chinese medicine antitumor formula.

## 2. Methods

### 2.1. Study Design

This retrospective study was conducted at Yantaishan Hospital, Yantai, Shandong, China, from February 2019 to February 2020 to explore the diagnostic value of serum Ang, VEGF, and CRP combined detection in the treatment of advanced renal carcinoma after being treated with the Chinese medicine antitumor formula. A total of 60 patients with advanced renal cancer were included in this study. This study was double-blind, and neither the subjects nor the researchers knew about the grouping of the tests. The study designer was accountable for arranging and controlling all the tests.

### 2.2. Object Recruitment

A retrospective analysis was performed on the data of patients admitted at Yantaishan Hospital, Yantai, Shandong, China, from February 2019 to February 2020, and patients were selected according to the following criteria: (1) Patients diagnosed with renal cancer by imaging and pathological examination, with stage III or IV according to Robson stage [17, 18]. (2) The patients treated in our hospital for the whole period without death cases, transfer to other hospitals, or cease of treatment. (3) The expected survival time was more than 3 months. (4) Patients with complete clinical data. (5) Patients over 18 years of age. (6) Patients with BMS, assessed without neurological abnormalities, and did not need to receive corticosteroid therapy. Patients were excluded according to the following criteria: (1) Unable to communicate with them due to hearing impairment, language barrier, unconsciousness, or mental illness. (2) Withdrawal from treatment, death, changes of treatment regimen, or loss of follow-up. (3) The presence of other serious diseases, such as liver, kidney, or cardiopulmonary dysfunction. (4) Abnormal hematopoietic function. (5) Severe infection exists.

### 2.3. Moral Considerations

This study was in accordance with the principles of the Declaration of Helsinki [19] and approved by the ethics committee of the Yantaishan Hospital, Yantai, China, and all the patients provided written informed consent for participation in the study.

### 2.4. Exit Test Criteria

If the following conditions occurred and the study group judged that it was unsuitable for continuing the study, the medical record form would be kept without data analysis: (1) Adverse events or serious adverse events occurred. (2) Deterioration of the condition during the test. (3) The subject has some serious coincidences or complications. (4) During the clinical trial, the object is unwilling to continue the clinical trial.

### 2.5. Methods

Sociodemographic data and clinical manifestation data were collected, and serological tests were performed before and after treatment with the Chinese medicine antitumor formula.

#### 2.5.1. Recipe Composition

Radix Pseudostellariae, Dahurian Patrinia Herb, Red rattan, yangtao actinidia root, Sealwort, Turtle shell, Turtle plate, and Bittersweet Herb, 15 g respectively, Atractylodes, *Magnolia officinalis*, Catechu, Celandine, and amomum, 10 g respectively, *Poria cocos* and Fructus Akebiae, 12 g respectively, Coix seed and *Oldenlandia diffusa*, 30 g respectively, Pulsatilla, 20 g, and liquorice, 3 g, were used. The Chinese medicine antitumor formula was decocted to 200 ml in warm water every day and divided into two doses for 14 days.

Before and after treatment, venous blood was collected from the patients for Ang, VEGF, and CRP detection. A total of 5 ml of venous blood was collected from the patients on an empty stomach in the morning, stood at room temperature for 30 min in anticoagulant tubes, and centrifuged at 3000 r/min for 10 min. The levels of Ang-1, Ang-2, VEGF, and CRP were determined by enzyme-linked immunosorbent assay (Beijing Kewei Clinical Diagnostic Reagent Co., LTD., S20060028).

### 2.6. Observation Criteria

(1) General Information: a general information extraction table was established, including the number of inpatients, name, sex, age, body weight, tumor stage, clinical classification, mean tumor diameter, main clinical symptoms, metastasis, and KPS score. (2) Changes of serum Ang, VEGF, and CRP before and after treatment. (3) Diagnostic value of serum Ang, VEGF, and CRP in patients treated with the Chinese medicine antitumor formula: sensitivity, specificity, and AUC of combined serum Ang, VEGF, and CRP were analyzed by ROC (95% CI).

### 2.7. Statistical Analysis

Data processing software was SPSS20.0, and the image rendering software was GraphPad Prism 7 (San Diego, USA). ROC was calculated by SPSS Statistics, and AUC (95% CI) was compared. The research included counting data and measurement data, utilizing the *χ*^2^ tests and *t*-test to analyze. *P* < 0.05 means the difference is statistically significant.

## 3. Results

### 3.1. Analysis of General Patient Data

There were 38 male and 22 female patients aged from 28 to 84 years, with an average age of 58.14 ± 3.68 years and an average bodyweight of 58.65 ± 1.65 kg. The tumor stages of all patients were stage III or IV, including 24 cases (40.0%) of stage III and 36 cases (60.0%) of stage IV. There were 52 cases of clear-cell carcinoma (86.7%), 7 cases of papillary carcinoma (11.7%), and 1 case of chromophobe carcinoma (1.7%). The main clinical symptoms of the patient were hematuria, abdominal mass, pain, an average tumor diameter of (9.67 ± 0.65) cm, and a KPS score of 74.68 ± 1.52. Lung metastasis was found in 18 (30.0%) cases, bone metastasis in 15 (25.0%) cases, liver metastasis in 10 (16.7%) cases, and brain metastasis in 2 (3.3%) cases (Table 1).

### 3.2. Analysis of Changes in Serum Ang, VEGF, and CRP before and after Treatment

**Figure 1:**
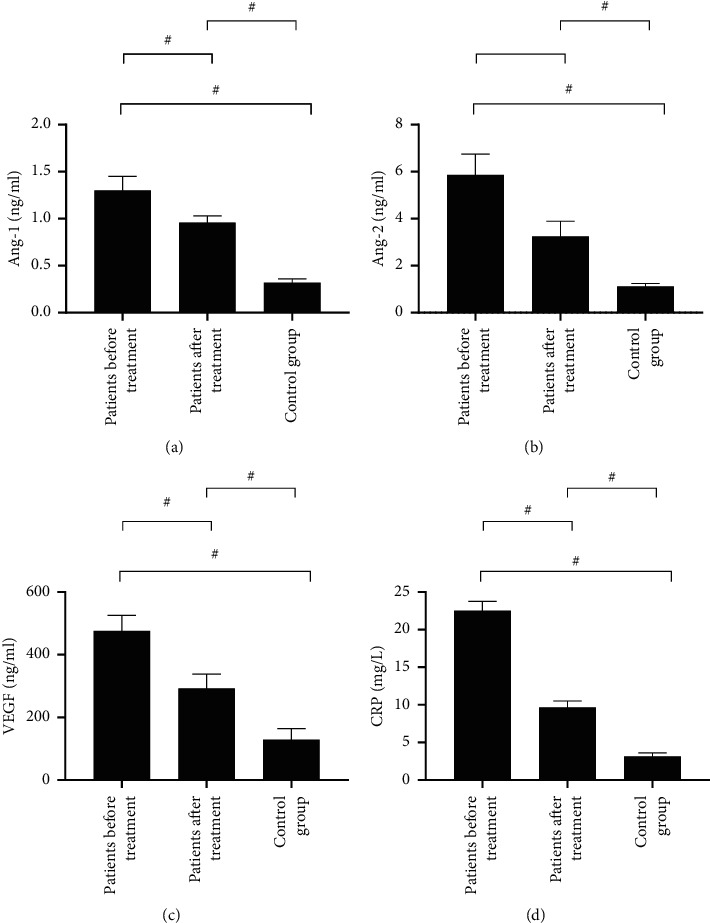
Analysis of changes in serum Ang, VEGF, and CRP before and after treatment (*x* ± *S*) vs. the control group, ^#^*P* < 0.01.

The levels of Ang-1 and Ang-2 after treatment were enormously lower than before treatment (0.96 ± 0.07 vs. 1.30 ± 0.15, *P* < 0.001 and 3.24 ± 0.65 vs. 5.86 ± 0.89, *P* < 0.001, respectively), but the levels of Ang-1 and Ang-2 before and after treatment were still significantly higher than those in the control group (1.30 ± 0.15 vs. 0.96 ± 0.07 vs. 0.32 ± 0.04, *P* < 0.001 and 5.86 ± 0.89 vs. 3.24 ± 0.65 vs. 1.12 ± 0.12, *P* < 0.001). The level of VEGF was tremendously lower after treatment than before (292.68 ± 45.62 vs. 475.68 ± 50.25, *P* < 0.001), but the levels of VEGF before and after treatment were still immensely higher than those in the control group (475.68 ± 50.25 vs. 292.68 ± 45.62 vs. 128.65 ± 35.44, *P* < 0.001). The level of CRP was remarkably lower after treatment than before (9.65 ± 0.87 vs. 22.54 ± 1.22, *P* < 0.001), but the level of CRP of patients before and after treatment was still obviously higher than that of the control group (22.54 ± 1.22 vs. 9.65 ± 0.87 vs. 3.16 ± 0.45, *P* < 0.001), as shown in Figure 1.

### 3.3. A Diagnostic Value Analysis of Serum Ang, VEGF, and CRP in Patients Treated with the Chinese Medicine Antitumor Formula

**Figure 2:**
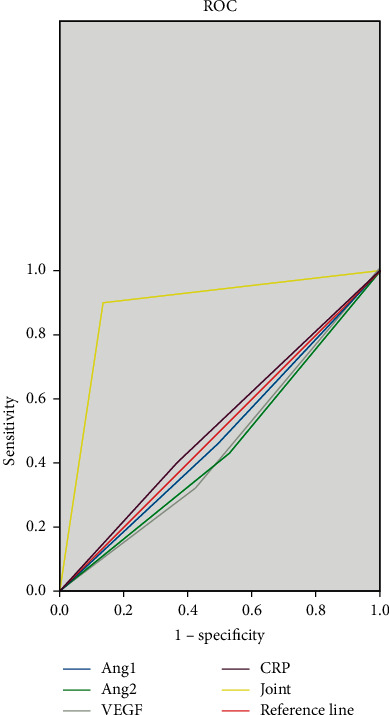
The diagnostic value of serum Ang, VEGF, and CRP before treatment. *Note*. The horizontal axis is 1-specificity, and the vertical axis is sensitivity. The blue line is the test result of Ang-1, the green line is the test result of Ang-2, the gray line is the test result of VEGF, the purple line is the test result of CRP, the yellow line is the test result of combined detection, and the red line is the reference line.

**Figure 3:**
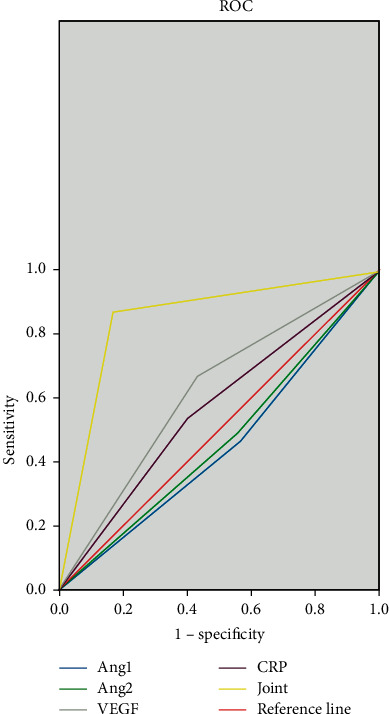
The diagnostic value of serum Ang, VEGF, and CRP after treatment. *Note*. The horizontal axis is 1-specificity, and the vertical axis is sensitivity. The blue line is the test result of Ang-1, the green line is the test result of Ang-2, the gray line is the test result of VEGF, the purple line is the test result of CRP, the yellow line is the test result of combined detection, and the red line is the reference line.

The sensitivity, specificity, and AUC (95%CI) of the combined detection of Ang, VEGF, and CRP before treatment were 86.7%, 90.0%, and 0.883 (0.817–0.950). The sensitivity, specificity, and AUC (95%CI) of the combined detection of Ang, VEGF, and CRP were 83.3%, 86.7%, and 0.850 (0.776–0.9524) after the treatment with the Chinese medicine antitumor formula, as shown in Figures 2 and 3.

## 4. Discussion

Renal cancer is a common malignant tumor of the urinary system, and its morbidity and mortality rank 14th and 16th, respectively, among malignant tumors globally [20]. The number of new patients with renal cancer is more than 400,000, and the number of deaths is more than 140,000 annually [21]. According to the data of the China Cancer Prevention and Control Research Office, the incidence of renal cancer in China is close to the average world level and is increasing year by year, with an average annual increase of about 6.0% [22, 23], indicating that it is a severe threat for Chinese residents. As the pathogenesis of renal cancer has not been clearly defined, the clinical treatment outcomes of renal cancer are not satisfactory, and immunotherapy in the past has significant side effects, and the remission rate is not high, accompanied by no significant improvement in the survival of patients [24, 25]. For patients with advanced renal cancer, chemotherapy and radiotherapy are the conventional treatment methods. However, patients with advanced renal cancer are usually accompanied by metastasis. Chemotherapy and radiotherapy cannot improve the 5-year survival rate, resulting in a poor prognosis. Research in recent years found that traditional Chinese medicine can effectively improve the immune status of patients and improve the overall curative effect, and the practice also confirmed that cancer patients prefer the combination of traditional Chinese and Western medicine treatment, indicating that there is a broad space for the application of traditional Chinese medicine treatment [26]. The Chinese medicine antitumor formula evaluated in this study includes the root of Kudanophora japonicus, Radix Pseudostellariae, Bittersweet Herb, Atractylodes, Celandine, *Poria cocos*, Fructus Akebiae, and *Oldenlandia diffusa*, which can effectively enhance the immune function of patients, make T lymphocytes kill mutant cells in time, induce cell autophagy, and inhibit the progression of cancer. Some scholars evaluated the Chinese medicine antitumor formula in colon cancer patients and found that the medicine could prolong the survival period of patients. Moreover, relevant reports showed that the inhibitory rate of *Oldenlandia diffusa* in the prescription was over 80% [27], so the Chinese medicine antitumor formula may be beneficial to alleviate the disease progression of patients with advanced renal cancer.

There are few studies that evaluated the treatment of advanced renal cancer with a Chinese medicine antitumor formula, so its actual effect is not authenticated. In this study, the reason why serum Ang, VEGF, and CRP were utilized as an indicator of its efficacy in this study is that the Chinese medicine antitumor formula can control cell proliferation by inhibiting vascular hyperplasia, while both Ang and VEGF are closely related to vascular hyperplasia. The VEGF gene is highly expressed in lung cancer, liver cancer, and other tumors, while Ang is closely related to the occurrence and development of tumors. Ashing Kimlin Tam et al. reported that the Yangtao Actinidia Root could regulate the proliferation and apoptosis of breast cancer cells through the VEGF signaling pathway [28]. Zhang et al. found that compound preparation Yangtao Actinidia Root could inhibit the growth of transplanted tumor in CT26 mice and control the expression level of VEGF [29]. In this study, there were significant differences in serum Ang and VEGF levels after treatment compared with before treatment, indicating that the renal cancer cells in the patient were controlled and the proliferation rate decreased significantly. In addition, CRP can also be considered to assess the prognosis of cancer patients. This indicator is not only an inflammatory indicator but also an immune indicator, which can activate complement, enhance cell phagocytosis, and maintain the normal function of the immune system. However, when patients' body is in stress conditions or diseases, CRP tends to rise; therefore, it is essential to maintain a rational CRP level. In this study, the CRP level of patients was improved after treatment, suggesting that the Chinese medicine antitumor formula has certain effects on the treatment of advanced renal cancer, and Ang, VEGF, and CRP can be considered as vital indicators to assess the disease progression in patients.

Among the patients included in this study, there were 52 cases (86.7%) of clear-cell carcinoma, 7 cases (11.7%) of papillary carcinoma, and 1 case (1.7%) of chromophobe cell carcinoma, with an average tumor diameter of (9.67 ± 0.65) cm. The sensitivity, specificity, and AUC (95% CI) of the combined detection of Ang, VEGF, and CRP before treatment were 86.7%, 90.0%, and 0.883 (0.817–0.950), while after treatment, the sensitivity, specificity, and AUC (95% CI) of the combined detection of Ang, VEGF, and CRP were 83.3%, 86.7%, and 0.850 (0.776–0.9524), suggesting that the combined detection of Ang, VEGF, and CRP has not only high diagnostic value for patients with advanced renal cancer before treatment but also good diagnostic value for patients after Chinese medicine antitumor formula treatment, which is conducive to the clinical judgment of the disease state of patients.

In conclusion, the combined detection of serum Ang, VEGF, and CRP has a potential diagnostic value for the Chinese medicine antitumor formula in treating patients with advanced renal cancer.

## Figures and Tables

**Table 1:** The demographic characteristics of patients.

Demographic characteristics (*n*)	Observation group (*n* = 60)
Male	38
Female	22
Age (‾*χ*±SD)	28–84 (58.14 ± 3.68)
Body weight (‾*χ*±SD)	58.65 ± 1.65

*Tumor stages*	
Stage III	24 (40.0%)
Stage IV	36 (60.0%)
Clear-cell carcinoma	52 (86.7%)
Papillary carcinoma	7 (11.7%)
Chromophobe carcinoma	1 (1.7%)
Tumor diameter	(9.67 ± 0.65)
KPS score	74.68 ± 1.52

*Tumor metastasis*	
Lung metastasis	18 (30.0%)
Bone metastasis	15 (25.0%)
Liver metastasis	10 (16.7%)
Brain metastasis	2 (3.3%)

## Data Availability

The data used to support the findings of this study are available from the corresponding author upon request.
